# Emerging prospects of vitamin D3 in metabolic syndrome: A proof of concept (POC) approach targeting inflammation

**DOI:** 10.17179/excli2020-2964

**Published:** 2020-11-08

**Authors:** Yogendra Singh, Gaurav Gupta, Ritu M. Gilhotra, Sachin Kumar Singh, Parteek Prasher, Anand Krishnan, Kamal Dua, Dinesh Kumar Chellappan

**Affiliations:** 1 Mahatma Gandhi College of Pharmaceutical Sciences, Sitapura, Jaipur, India; 2 School of Pharmacy, Suresh Gyan Vihar University, Mahal Road, Jagatpura, Jaipur, India; 3 School of Pharmaceutical Sciences, Lovely Professional University, Phagwara, Punjab, 144411, India; 4 Department of Chemistry, University of Petroleum & Energy Studies, Dehradun, 248007, India; 5 Department of Chemical Pathology, School of Pathology, Faculty of Health Sciences and National Health Laboratory Service, University of the Free State, Bloemfontein, South Africa; 6 Discipline of Pharmacy, Graduate School of Health, University of Technology Sydney, Ultimo, NSW 2007, Australia; 7 Department of Life Sciences, School of Pharmacy, International Medical University, Kuala Lumpur 57000, Malaysia

## ⁯

***Dear Editor,***

Metabolic syndrome (MetS) comprises of a group of associated disease conditions. These primarily include insulin resistance, dyslipidemia, and high blood pressure. Visceral adiposity majorly contributes to the development and pathogenesis of the metabolic syndrome. Most of all of these factors contribute to increased cardiovascular risks. MetS is the principal precursor for type 2 diabetes mellitus. It raises the potential risk of inflammatory cardiovascular disease (CVD) by 2 folds and further causes a 1.5-fold increase in the risk of mortality (Wilson et al., 1999[22]). Therefore, all the components about MetS pose a considerable risk, especially in the pathophysiology of CVD. It is crucial to recognize and treat each contributing factor to reduce the risks of CVD. The primary mechanism leading to the development and pathogenesis of MetS is chronic inflammation and other contributing inflammatory elements. In this short letter, we have summarized notable findings from recent studies that may provide crucial insights concerning the underlying inflammatory mechanisms that lead to the development of MetS. Moreover, based on such mechanisms, we have identified potential treatment strategies, including vitamin D, which could be a promising agent in the long-term management of inflammation and its associated conditions in MetS. 

Visceral adiposity, excessive intake of a high-fat diet, and defect in fat metabolism *via* overstimulation of RAAS and sympathetic system are the major causative factors leading to the pathogenesis of insulin resistance. These pathogenic factors further form the common and essential link between inflammation and MetS. Several mechanisms are currently known, that show the progression of insulin resistance to chronic inflammation, which further progresses to CVD in MetS (Prasad and Quyyumi, 2004[16]). A substance called monocyte chemo-attractant protein-1 (MCP-1) is produced and secreted by the adipocytes. Moreover, they also secrete other cytokines, namely, TNF-α and IL-6. These substances predominantly cause infiltration of macrophages into adipose tissues (Donath and Shoelson, 2011[7]). As a result, these events lead to the mediation of downstream pathways namely, IKK and JNK that produce defective hyperphosphorylation of IRS-1 resulting in the development of insulin resistance by disturbing the PI3K/AKT signaling. Moreover, adipose tissue also regulates Adiponectin, a contributing factor for insulin resistance, *via* maintaining the homeostasis of inflammatory and anti-inflammatory cytokine components, IL-6/TNF-α and IL-10 respectively. Furthermore, JNK and IKK activation may lead to the upregulation of NF-κB and AP-1 activation. This may result in the production of inflammatory cytokines (Fujioka et al., 2004[9]). Also, inflammasomes are mediated by adipose tissue-derived components like free fatty acid, causing the activation of nods like receptors that leads to the formation of IL-1b and IL-18. These are products of cleavage resulting from the action of caspase-1 of pro-IL-1b and pro-IL-18 (Welty et al., 2016[21]). Excessive high fat diet further triggers the inflammatory cascade and insulin resistance *via* the expression of PLA2 -COX/LOX associated synthesis of PGs and LTs respectively. High fat may also recruit neutrophil-mediated IL-6 and TNF-α release. Besides, LT's associated chemotactic factor (LTB4) release, further upregulates NF-κB mediated inflammatory cytokines (Horrillo et al., 2010[13]). Dyslipidemia in MetS leads to high TG and low HDL cholesterol levels, which result in the production of ApoB-100/ApoC-III fatty acid content. It is reported that ApoC-III plays a imperative role in the progression of inflammation. Furthermore, it is also reported to activate the TLR2 signaling pathway in experimental animals, thereby, serving as an inflammatory mediator (Gupta et al., 2014[12]). The resulting cascade of events result in the upregulation of NF-κB leading to the upregulation of cytokine production. These mechanisms essentially cause insulin resistance, atherosclerosis and thrombosis.

Dietary modifications, weight management, exercise along with lifestyle therapy are the key principles outlining the effective management of MetS. However, various pharmacological approaches are also available for controlling the co-morbidities. These include anti-diabetic drugs like metformin, glucagon-like-peptide 1 (GLP-1) agonists, other oral hypoglycemic agents like sodium-glucose co-transporter 2 (SGLT2) inhibitors, inhibitors of dipeptidyl peptidase 4 (DPP4) enzyme, RAAS blockers (ACE inhibitors and AR blockers), hypolipidemic agents (Statins and PPAR-α), K^+ ^sparing diuretics (aldosterone antagonists) and omega 3 fatty acids (Aguilar-Salinas and Viveros-Ruiz, 2019[1]). Furthermore, these pharmacological interventions have notable disadvantages and do not effectively suppress the risks of disease progression. Meta-analysis data from 13 major statin trials, over 4 years, have shown a 9 % increase in the development of diabetes when compared with placebo (Sattar et al., 2010[17]).

**Figure 1:**
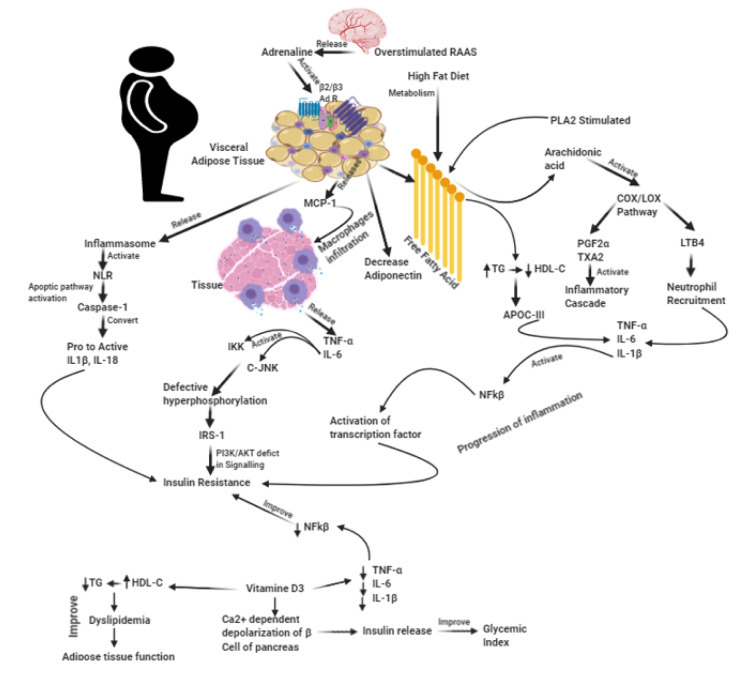
Role of vitamin D3 in inflammatory metabolic syndrome

In this short communication, we have attempted to explore the robust evidence-based effect of vitamin D_3 _(VitD_3_) / cholecalciferol supplementation on the inflammation cascade and insulin resistance, which may alter the CVD outcomes in patients with MetS. VitD_3_ deficiency is increasingly recognized as a major health problem across the globe. In India alone, the prevalence rates of VitD_3_ deficiency is reported to be as high as 94 % in urban areas. This could be essentially due to poor exposure to sunlight and a sedentary lifestyle, mainly confined to their homes or workplaces (Vupputuri et al., 2006[20]). There are several studies, conducted across the globe, which connect VitD_3_ deficiency to inflammatory conditions like MetS. A clinical study conducted among 157 Asian Indians revealed that VitD_3_ deficiency was found in 73.25 % of the individuals. These subjects also had prediabetes. A higher insulin resistance score was observed in subjects having lower VitD3 levels (Dutta et al., 2013[8]). A recent 18-month randomized control trial published in Nature Scientific Reports, revealed a significant decrease in parameters namely, fasting blood sugar levels, glycated hemoglobin, and subcutaneous fat with VitD_3_ administration (Bhatt et al., 2020[4]). Moreover, another trial conducted on migrant South Asian women, who had insulin resistance revealed valuable data on VitD_3_ administration. The findings revealed that improvement in VitD_3_ status reduced insulin resistance and increased insulin sensitivity (Gupta et al., 2020[11]; von Hurst et al., 2010[19]). VitD_3_ also regulates the action of a key substance called calbindin. Calbindin is a protein that is usually seen in the pancreatic cells. Physiologically, it binds to calcium within the cells and regulates insulin secretion through cellular depolarization (Kadowaki and Norman, 1984[14]). VitD3 supplementation further normalizes and regulates glucose triggered secretion of insulin. It also modulates the generation and physiology of cytokines, thereby enhancing β-cell survival (Cade and Norman, 1987[5]; Singh et al., 2017[18]). VitD_3_ in diabetes has also demonstrated enhanced action of insulin, in terms of transporting glucose and reducing the overall levels of cellular inflammation through the regulation of IL-6, TNF-α, IL-10, hsCRP. It further maintains the relative levels of IL-6 to IL-10 along with NFκB transcriptional activity (De Vita et al., 2014[6]; Pittas et al., 2007[15]). The antihypertensive function of VitD_3 _is proposed through suppression of the renin-angiotensin pathway with its anti-endothelial stiffness effect, followed by secondary hyperparathyroidism prevention. However, progressive renal dysfunction in diabetes mellitus (DM) after VitD_3_ administration has resulted in increased intrarenal vascular calcification. This highlights the possible harmful effects of long-term supplementation of VitD_3_ (Almeida et al., 2020[3]; Gupta et al., 2012[10]). However, lower or insufficient VitD3 in male subjects led to increased levels of triglycerides and a decrease in their HDL-C levels. Furthermore, crucial findings from another clinical study showed that HDL-C levels were comparatively boosted up in patients who were administered with optimal doses of VitD3 (Alkhatatbeh et al., 2019[2]) (Figure 1(Fig. 1)).

As the concluding remark, we provide the proof-of-concept for VitD_3_, which significantly acts as a key factor in the suppression of pro-inflammatory elements, thereby improving insulin resistance that modifies the consequences of CVD in MetS, and thus should be included in the treatment regimen.

## Conflict of interest

The authors declare no conflict of interest.
